# A Loop-Mediated Isothermal Amplification Assay for Rapid Detection of Cyprinid Herpesvirus 2 in Gibel Carp (*Carassius auratus gibelio*)

**DOI:** 10.1155/2014/716413

**Published:** 2014-01-19

**Authors:** Hui Zhang, Lingbing Zeng, Yuding Fan, Yong Zhou, Jin Xu, Jie Ma

**Affiliations:** Division of Fish Disease, Yangtze River Fisheries Research Institute, Chinese Academy of Fishery Sciences, Wuhan, Hubei 430223, China

## Abstract

A rapid and sensitive loop-mediated isothermal amplification (LAMP) assay for Cyprinid herpesvirus 2 (CyHV-2) detection in gibel carp was developed. Following cloning and sequencing of the putative DNA helicase gene of CyHV-2 isolate from China, a set of four specific primers was designed based on the sequence. The MgCl_2_ concentration and the reaction temperature were optimized to 6 mM, 64°C, respectively. LAMP products were detected by visual inspection of a color change due to addition of SYBR Green I stain. The specificity and sensitivity of the LAMP assay were determined. No cross-reaction was observed with other fish DNA viruses including eel herpesvirus, koi herpesvirus, and Chinese giant salamander iridovirus. The LAMP assay was found to be equally sensitive as nested PCR. A comparative evaluation of 10 fish samples using LAMP and nested PCR assays showed an overall correlation in positive and negative results for CyHV-2. These results indicate that the LAMP assay is simple, sensitive, and specific and has a great potential use for CyHV-2 detection in the laboratory and field.

## 1. Introduction

Herpesviral hematopoietic necrosis (HVHN), caused by Cyprinid herpesvirus 2 (CyHV-2), is a disease of goldfish *Carassius auratus auratus* (Linnaeus, 1758) that was first reported in juvenile goldfish in Japan in the spring and autumn of 1992 and the spring of 1993 and later in Australia, Taiwan, and the USA [1, 2]. The CyHV-2 has been classified as a member of the genus *Cyprinivirus*, the family Alloherpesviridae in the Herpesvirales order, and its complete genome has been sequenced (GenBank accession JQ815364).

Molecular tools such as polymerase chain reaction (PCR) and quantitative real-time PCR have been established for the detection and quantification of CyHV-2 [1–7]. However, these methods require the use of expensive equipment and costly consumables, which have limited their application. Loop-mediated isothermal amplification (LAMP) is a technique that has been developed to amplify nucleic acids with high specificity, sensitivity, and rapidity under isothermal conditions [8]. In aquaculture, LAMP assays have been developed to detect fish and shellfish pathogens including aquatic DNA viruses such as red seabream iridovirus (RSIV) [9], white spot syndrome virus (WSSV) [10–14], koi herpesvirus (KHV, CyHV-3) [15–19], infectious hypodermal and hematopoietic necrosis virus (IHHNV) [20–22], hepatopancreatic parvovirus (PmDNV) [23], Singapore grouper iridovirus (SGIV) [24], acute viral necrobiotic virus (AVNV) [25], turbot reddish body iridovirus (TRBIV) [26], infectious spleen and kidney necrosis virus (ISKNV) [27], lymphocystis disease virus (LCDV) [28], ostreid herpesvirus 1 (OsHV-1) [29], and soft shelled turtle iridovirus (STIV) [30]. Therefore, LAMP has been a useful tool for the diagnosis of aquatic animal diseases.

Gibel carp (*Carassius auratus gibelio*) is a major species of aquaculture in China, which has been widely cultured in almost the whole country. Recently, an epizootic with severe mortality has emerged in cultured gibel carp in China and caused huge economic loss. The causative pathogen was isolated and identified as Cyprinid herpesvirus 2 (CyHV-2) by means of experimental infection, electron microscopy, cell culture, PCR assay, and sequence alignment [31]. In this study, a highly sensitive and specific diagnostic method based on the loop-mediated isothermal amplification (LAMP) for CyHV-2 detection in gibel carp was developed following cloning and sequencing of the DNA helicase gene of CyHV-2. Meanwhile, the reaction conditions were optimized and the specificity and sensitivity of the LAMP assay were assessed. This study represents the first report of LAMP assay for CyHV-2 detection in cultured gibel carp, which has great potential use in both laboratory and field diagnosis of the disease.

## 2. Materials and Methods

### 2.1. Viruses, Fish Samples, and Preparation of DNA Template

Cyprinid herpesvirus 2 (CyHV-2), eel herpesvirus (AngHV-1), koi herpes virus (KHV, CyHV-3), and Chinese giant salamander iridovirus (GSIV) were isolated and kept in our laboratory.

Diseased gibel carp (15–28 cm in length) were obtained from the farms in Jiangsu province, China in May 2012. Healthy fish were obtained from the experimental station, Yangtze River Fisheries Research Institute, Chinese Academy of Fishery Sciences.

The spleen and kidney of the diseased gibel carp with severe hemorrhagic symptoms were collected and homogenized on ice in DPBS (Sigma, USA) at a ratio of 1 : 5 w/v. After being frozen-thawed twice, the homogenate was centrifuged at 4,500 ×g for 30 min at 4°C (Sigma 3K15). 250 *μ*L of supernatant was used for DNA extraction with a commercial kit (Viral DNA Kit, OMEGA, USA) according to the manufacturer's protocol. The final elution of viral DNA was in 100 *μ*L sterile ddH_2_O and stored at −20°C for use.

### 2.2. Primers Design

A PCR primer set (JF/JR) was designed to amplify the complete cds of the viral putative DNA helicase gene (GenBank accession EU349287). Then nested PCR primers (JF1/JR1, JF2/JR2) were designed for detection (GenBank accession KC245087) by software Primer Premier 5.0.

**Figure 1:**
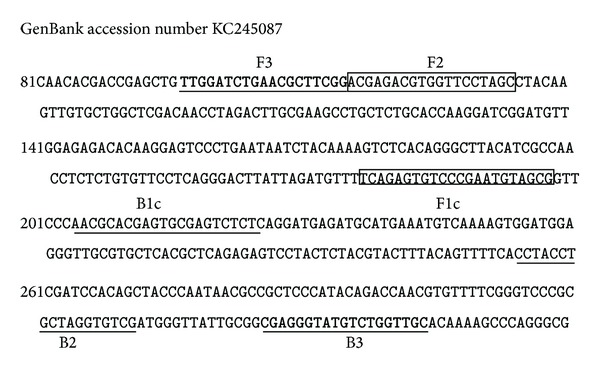
The nucleotide sequence of partial CyHV-2 DNA helicase gene used to design inner and outer primers for LAMP.

A set of four LAMP primers (JF3 and JB3, JBIP and JFIP) recognizing six distinct regions in the viral putative DNA helicase gene sequence (GenBank accession KC245087) was designed by using the Primer Explorer version 4 (http://primerexplorer.jp/elamp4.0.0/index.html). The primers' sequences and the locations were indicated in Figure 1. All primers were synthesized (Invitrogen, Shanghai, China) and are shown in Table 1.

### 2.3. Cloning and Sequencing of the Putative DNA Helicase Gene of CyHV-2

The putative DNA helicase gene of CyHV-2 was amplified by PCR. The reaction mixture contained 0.2 *μ*M each of JF and JR, 0.2 mM of dNTP mix, 1× PCR buffer, 2.5 U of Taq DNA polymerase (Takara, Dalian, China), 5 *μ*L DNA template in a final volume adjusted to 25 *μ*L with sterile ddH_2_O. The PCR thermal cycling protocol was 5 min at 95°C followed by 30 cycles of 95°C for 45 s, 56°C for 45 s, 72°C for 60 s, and a final extension at 72°C for 10 min ending in 4°C hold. PCR product was purified and cloned into the pMD18-T Vector (Takara, Dalian, China) according to the manufacturer's protocol; the recombinant plasmid was sequenced (Sangon, Shanghai, China).

### 2.4. Nested PCR Detection

Nested PCR was carried out using the primers designed. The first PCR was carried out with an outer primer set (JF1 and JR1), yielding a PCR product with 716 bp. The nested PCR was performed with an inner primer set (JF2 and JR2), yielding a PCR product with 357 bp.

The PCR reactions contained 0.2 *μ*M each of primers, 0.2 mM of dNTP mix, 1× PCR buffer, 2.5 U of Taq DNA polymerase (Sangon, Shanghai, China), 5 *μ*L DNA template, and sterile ddH_2_O in a final volume of 25 *μ*L. Amplification conditions were as follows: 95°C for 3 min, then 35 cycles of denaturation at 95°C for 30 s, annealing at 55°C for 30 s, extension at 72°C for 50 s, followed by a final elongation at 72°C for 5 min.

0.5 *μ*L of the first PCR product was used as DNA template for the nested PCR amplification using the same conditions as stated above. PCR products were electrophoresed on a 2% (w/v) ethidium bromide-stained agarose gel in Tris-boric acid-EDTA (TBE) buffer.

### 2.5. Optimization of LAMP Reaction Conditions

To optimize the LAMP reaction, different reaction temperatures and MgCl_2_ concentrations were tested. The LAMP reactions were performed using a heating block set at 60, 61, 62, 63, 64, and 65°C for 60 min, respectively. The LAMP reactions were incubated in a heating block set at 64°C for 60 min, containing 0, 2, 4, 6, 8, and 10 mM MgCl_2_, respectively.

The reaction mixture (25 *μ*L) contained 1.6 *μ*M each of inner primer (JBIP and JFIP), 0.2 *μ*M each of outer primer (JF3 and JB3), 1 mM of dNTP mix, 0.5 M betaine (Sigma, USA), 6 mM MgCl_2_, 8 U Bst DNA polymerase large fragment (New England Biolabs, USA), 1× the supplied buffer, and 5 *μ*L template DNA that was heated at 95°C for 5 min. Sterile ddH_2_O was included as negative control. The products were analyzed by 2% agarose gel electrophoresis.

### 2.6. Detection of LAMP Products

After LAMP reaction, white turbidity of the reaction mixture by magnesium pyrophosphate (by-product of LAMP) was inspected. Visual inspection of LAMP amplification was carried out by mixing 4 *μ*L products with 6 *μ*L 100-fold diluted original SYBR Green I (Molecular Probes, Inc.). The color change in the tubes was examined under natural light with the naked eye. Green fluorescence was observed clearly in the positive reaction, whereas it remained original orange in the negative reaction. The mixture was also examined under UV light (302 nm). Reaction products were also analysed by gel electrophoresis, 5 *μ*L aliquots were analysed on a 2% agarose gel with ethidium bromide.

### 2.7. Specificity of the LAMP Assay

The specificity of the LAMP assay to amplify only CyHV-2 DNA was tested against DNA extracted from other viruses including eel herpesvirus (AngHV-1), koi herpesvirus (KHV, CyHV-3), and Chinese giant salamander iridovirus (GSIV). Sterile ddH_2_O was used as a negative control. The LAMP products were analyzed by 2% agarose gel electrophoresis.

### 2.8. Sensitivity of the LAMP Assay

To determine the lower detection limit of the LAMP assay, 5 *μ*L of CyHV-2 DNA extracted from diseased fish was 10-fold serially diluted and subjected to CyHV-2 LAMP assay in comparison with nested PCR assay. The LAMP reaction was performed at 64°C for 60 min. Nested PCR was also performed for the same samples simultaneously. Amplification products were analyzed by agarose gel electrophoresis.

### 2.9. Evaluation of the LAMP Assay

The use of CyHV-2 LAMP assay to detect CyHV-2 in clinical specimens was evaluated by testing a total of 5 CyHV-2 infected and 5 healthy fish, which were tested previously by nested PCR. Amplification products were analyzed by agarose gel electrophoresis.

## 3. Results

### 3.1. Analysis of the Putative DNA Helicase Gene of CyHV-2

Analysis of the viral DNA helicase gene obtained in this study showed that it was almost identical with that of the Japanese strain of CyHV-2. When the sequence alignment (BLAST) was performed, 99% nucleotide identity to the published CyHV-2 sequences (GenBank accession JQ815364, EU349287) was observed. However, these differences resulted in no alteration of the amino acid sequence (GenBank accession JQ815364), but only one amino acid alteration (GenBank accession EU349287). The sequence of the DNA helicase gene of CyHV-2 isolated from gibel carp in this study was deposited in GenBank (accession number KC245087).

### 3.2. Optimization of the LAMP Reaction Conditions

**Figure 2:**
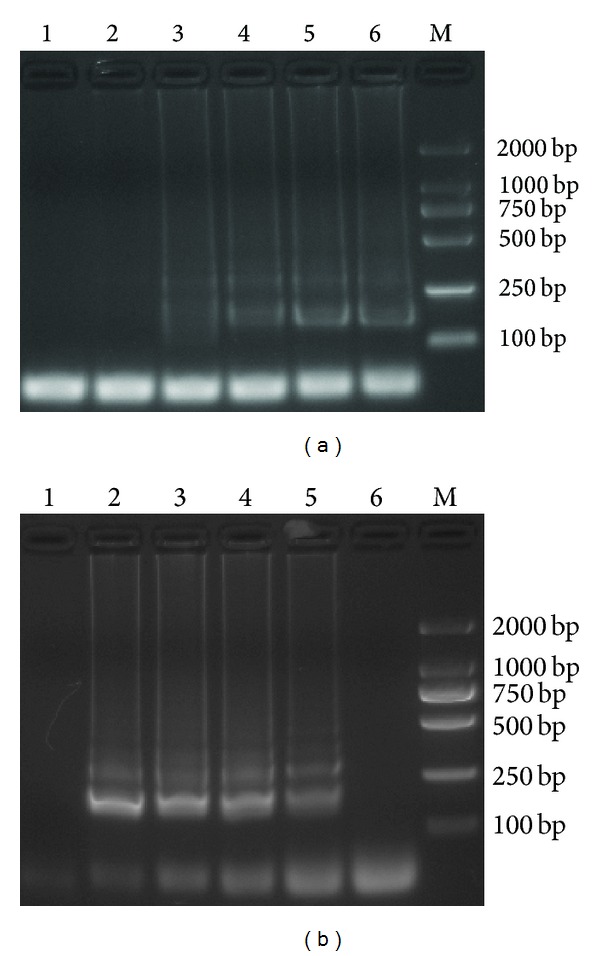
Optimization of the LAMP reaction conditions. (a) Reaction temperature: 1–6: 60°C, 61°C, 62°C, 63°C, 64°C, and 65°C, M: DL2000 DNA marker. (b) MgCl_2_ concentration: 1–6: 0 mM, 2 mM, 4 mM, 6 mM, 8 mM, and 10 mM MgCl_2_: M: DL2000 DNA marker.

The optimal LAMP reaction temperature revealed that although detectable results were observed at 62–65°C, the LAMP product amplified at 64°C exhibited slightly larger amounts of DNA as compared to others (Figure 2(a)).

The optimal MgCl_2_ concentration for LAMP showed that tests with 2–8 mM MgCl_2_ gave detectable results but that the clearest bands were obtained with 6 mM MgCl_2_ (Figure 2(b)).

Thus, the optimal reaction conditions were 6 mM MgCl_2_, 64°C. In addition, because loop primers were not used, 60 min was chosen arbitrarily as the assay time, consistent with the classic report about LAMP [8]. These conditions were used in the subsequent experiments.

### 3.3. Specificity of the LAMP Assay

**Figure 3:**
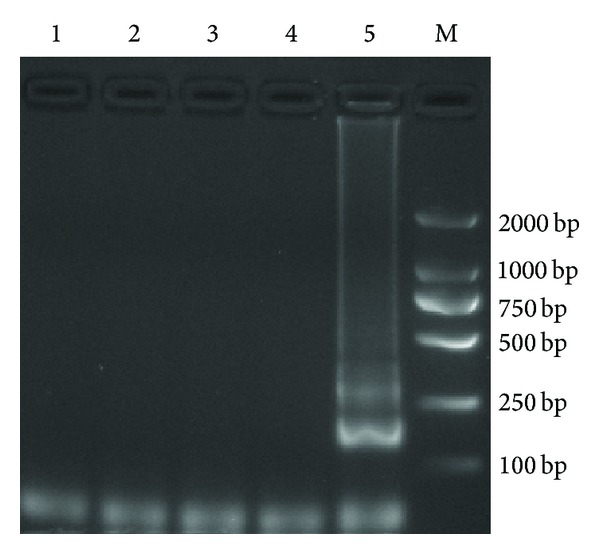
Specificity of the LAMP assay. 1: negative control; 2: GSIV; 3: AngHV-1; 4: KHV; 5: CyHV-2; M: DL2000 DNA marker.

The LAMP assay result showed that a typical ladder-like pattern was observed only when the CyHV-2 DNA was present. There were no amplification products detected with eel herpesvirus (AngHV-1), koi herpesvirus (KHV, CyHV-3), or Chinese giant salamander iridovirus (GSIV) genomic DNA (Figure 3).

### 3.4. Sensitivity of the LAMP Assay

**Figure 4:**
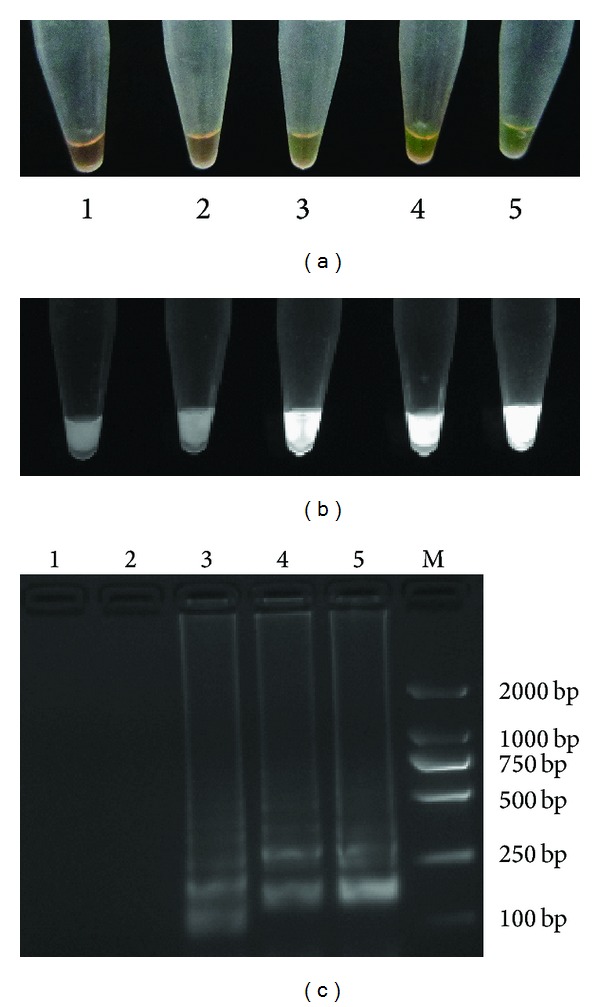
Sensitivity of the LAMP assay. Visual inspection in daylight (a), under UV light (b), and with 2% agarose gel electrophoresis (c). 1: negative control; 2: 10^−4^ dilution; 3: 10^−3^ dilution; 4: 10^−2^ dilution; 5: 10^−1^ dilution; M: DL2000 DNA marker.

**Figure 5:**
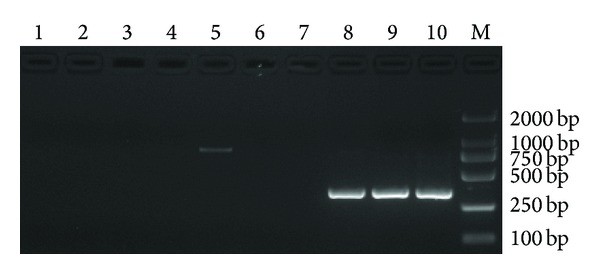
Sensitivity of the nested PCR assay. 1–5: the first PCR products; 6–10: the second PCR products. 1, 6: negative control; 2, 7: 10^−4^ dilution; 3, 8: 10^−3^ dilution. 4, 9: 10^−2^ dilution; 5, 10: 10^−1^ dilution; M: DL2000 DNA marker.

The reaction was tested using 5 *μ*L of the 10-fold serial dilutions of CyHV-2 DNA extracted from positive clinical samples and compared against results from the nested PCR assay. When LAMP reactions were carried out at 64°C for 60 min, the LAMP could result in amplification of up to 10^−3^ dilution, comparable to nested PCR (Figures 4 and 5). These observations also showed that the results of the LAMP assay determined by the naked eye or by UV spectroscopy agreed with those obtained by gel electrophoresis.

### 3.5. Applicability of the LAMP Assay

When fish samples were tested, the LAMP assay results correlated strongly with nested PCR results for CyHV-2 detection (data not shown). The positive samples tested by nested PCR also gave positive reactions with LAMP, and no sample that was negative by nested PCR tested positive with the LAMP assay, which proved the applicability of the assay in CyHV-2 disease diagnosis.

## 4. Discussion

The most classic technique for detection of viral pathogens is virus isolation by cell culture, but this technique depends on the subculture of cells and virus and is time consuming. It is reported that the CyHV-2 is very difficult to be cultured in cells [1–4, 31], so the PCR and real-time PCR assays are mainly used for detection and quantification of CyHV-2 [1–7]. In this study, the LAMP assay for CyHV-2 detection was developed successfully.

The LAMP reaction conditions, including MgCl_2_ concentration and reaction temperature, were optimized first in the study. The optimal conditions of LAMP reaction for the detection were determined at 64°C for 60 min with 6 mM MgCl_2_.

Specificity and sensitivity are the most important parameters to evaluate an assay. LAMP assays carried out using DNA templates from AngHV-1, KHV (CyHV-3) and GSIV gave no positive results (i.e., no amplification). The results indicated that the LAMP assay was specific for CyHV-2. The detection limit of the LAMP assay was determined by amplification of 10-fold serial dilutions. The results obtained by LAMP gave comparable sensitivity to those of nested PCR at 10^−3^ dilution, which was in agreement with previous reports [11, 22, 23].

Use of SYBR Green I for visual inspection of LAMP results was a simple and superior technique, with no gel electrophoresis and staining with ethidium bromide required. Positive and negative reactions showed distinctly different colors in daylight on a black background [26]. Although quantitative detection is difficult, the eye inspection was simple and rapid. Therefore, it may facilitate the application of LAMP, especially as a field test.

According to known sequences, the viral DNA helicase gene used as the target in the LAMP assay is relatively well conserved, which was useful for detection of all CyHV-2 isolates. Our final goal is to exploit a simple and rapid diagnostic kit for diagnosis and monitoring of CyHV-2 in fish farms, as it does not require special equipment. Further studies are considered, such as a simple DNA extraction method by tissue boiling.

In conclusion, a simple, rapid, and sensitive LAMP assay to detect CyHV-2 in fish was developed and validated. In this simple diagnostic protocol, the reaction was carried out in a single tube and incubated for 60 min in a water bath or a heating block at 64°C. LAMP products could be detected by the naked eye with the aid of SYBR Green I stain, which could facilitate field implementation of the LAMP assay.

## Figures and Tables

**Table 1:** Primer names and sequences used in the study.

Primer	Sequence (5′→3′)	Position
JF	ATGTGCAACGTGACGGCGAGT	1–21
JR	CTACCGTCTTTTAGGG	1446–1431
JF1	TGAAATGTCAAAAGTGGATGG	239–259
JR1	TATTCCCAGACAGCCTTCAAA	954–934
JF2	GAACACCGCTGCTCATCATC	323–342
JR2	ACTCTTCGCAAGTCCTCACC	679–660
JF3	TTGGATCTGAACGCTTCGG	97–115
JB3	CGTTGGTCTGTATGGGAGC	286–304
JFIP	GCGATGTAAGCCCTGTGAGACTTTTTACGAGACGTGGTTCCTAGC	176–197/TTTT/116–134
JBIP	AACGCACGAGTGCGAGTCTCTTTTGCTGTGGATCGTCCATCC	204–223/TTTT/254–271
